# Pre-qualifications, learning strategies, and study satisfaction among medical students: insights from a multicenter German study

**DOI:** 10.1080/10872981.2026.2694212

**Published:** 2026-06-25

**Authors:** Carla Schröpel, Anne Herrmann-Werner, Teresa Festl-Wietek, Tim Wittenberg, Sabine C. Herpertz, Katrin Schüttpelz-Brauns, Andrea Heinzmann, Tobias Böckers, Stephan Zipfel, Rebecca Erschens

**Affiliations:** a University Medical Hospital Tübingen, Internal Medicine, Department of Psychosomatic Medicine and Psychotherapy, Tübingen, Germany; b TIME -Tübingen Institute for Medical Education, Medical Faculty, University of Tübingen, Tübingen, Germany; c heiTEST-Department, Medical Faculty Heidelberg, University of Heidelberg, Germany; d Department of General Psychiatry, Centre for Psychosocial Medicine, University of Heidelberg, Heidelberg, Germany; e Medical Education Research Department, Division for Study and Teaching Development, Medical Faculty Mannheim at Heidelberg University; f Albert-Ludwigs-University Freiburg, Medical Faculty, Office of the Dean of Studies, Freiburg, Germany; g Office of the Dean of Studies, Medical Faculty, Ulm University, Ulm, Germany; h Deanery of Students’ Affairs, University's Faculty of Medicine, Tübingen, Germany

**Keywords:** Medical students, study satisfaction, learning strategies, part-time employment, prior qualifications

## Abstract

**Introduction:**

Although medical students with pre-medical qualifications (e.g., paramedic training) may enhance diversity within the student body, they have received little attention in international research. Their prior experience may influence study satisfaction through different learning strategies or working alongside their studies. This study investigates how pre-qualifications relate to three dimensions of study satisfaction (content, conditions, and coping with study load) and examines whether deep-processing learning strategies (elaboration and critical thinking) and part-time employment help explain these associations.

**Method:**

The authors conducted a cross-sectional survey at five German medical schools across different stages of study (3rd, 6th and 10th semester, final year). A structural equation model tested (a) whether pre-qualifications (i.e., academic degree, vocational training in the medical field) moderated the relationship between deep-processing strategies and study satisfaction and (b) whether part-time employment mediated the relationship between pre-qualifications and study satisfaction, controlling for age, gender, undergraduate GPA, semester, and medical school.

**Results:**

Associations between deep-processing strategies and satisfaction did not differ by pre-qualifications. Regardless of students' pre-qualifications, elaboration was positively (ß = .51) and critical thinking negatively (ß = −.31) associated with satisfaction with study contents. Students with vocational training or prior academic degree more often worked part-time to finance studies; part-time employment was associated with lower satisfaction with study conditions (ß = −.07) but higher satisfaction with coping with study load (ß = .08). Indirect effects via part-time employment were significant but small.

**Discussion:**

The findings suggest that elaboration may be a particularly relevant learning strategy for satisfaction with study contents, regardless of students' prior professional or academic qualifications. The negative association between critical thinking and satisfaction highlights the complexity of students’ perceptions of study contents. Although associations involving part-time employment were small, the findings contribute to a better understanding of students with professional and academic pre-qualifications and may inform efforts to support increasingly diverse medical student populations.

## Introduction

Medical students' satisfaction with their studies is important not only for their personal development, but also for quality assurance in medical education. It is closely tied to students' mental health [1,2], self-efficacy, and optimistic outcome expectations about their studies [3]. At the institutional level, higher satisfaction contributes to lower dropout rates and enhances the attractiveness of degree programmes [4,5]. Given these associations, a better understanding of the specific factors influencing study satisfaction in medical education is crucial. Meta-analytic findings could identify the university environment, service quality, and perception of the resources provided as possible antecedents of study satisfaction [5]. Additionally, student factors such as intrinsic motivation for choosing the programme of study and conscientiousness contribute to higher levels of satisfaction [6]. However, these findings are derived from non-medical student populations, highlighting the need for more research within medical education.

Internationally, standardised instruments have been developed to measure satisfaction across different educational contexts [7]. One prominent and validated instrument used in Germany is the Study Satisfaction Questionnaire [8]. Based on the context of job satisfaction, the authors defined study satisfaction as students' attitudes towards their studies [8,9]. Factor analyses of the short version indicate three separate dimensions: satisfaction with the study contents, satisfaction with the conditions and satisfaction with coping with study load [8], enabling a differentiated assessment of satisfaction levels. Although reference values exist for medical students [8], they should not be viewed as a homogeneous group. Differences in prior practical experiences may influence satisfaction, yet little is known about the satisfaction levels of medical students with *professional or academic pre-qualifications* [10]. In the present study, this includes students with (i) completed vocational training in the medical field, (ii) prior academic degree, or (iii) completed voluntary service. Internationally, medical schools have increasingly sought to diversify their student populations through widening participation initiatives and alternative admission pathways [11–13]. These efforts aim to broaden access for applicants from diverse educational, socioeconomic, and cultural backgrounds, including so-called 'non-traditional' students [14–17]. Germany's medical education system provides an interesting case in point. Although cognitive abilities are still the most decisive admission criterion, a significant proportion of students with relevant prior practical experience (e.g., vocational training in the medical field, voluntary service) are accepted [10,18,19]. In this context, the targeted admission of applicants who did not proceed directly to medical school after secondary education may likewise be considered a widening participation measure, as it facilitates access for individuals with more diverse educational, professional, and biographical backgrounds [20]. While these medical students may enrich classrooms through their prior experiences, they may also require different learning environments and might thus differ in satisfaction compared to students entering directly after high school. Also, as medical studies represent a second educational pathway, students with vocational training or a prior academic degree are typically older and may receive less financial support from their parents, [18,21]. Although tuition fees are charged to second-degree students in only a few German federal states, students must nevertheless cover their living expenses, and studies indicate lower levels of academic performance and study satisfaction among those who are employed [22,23]. Balancing financial demands through part-time employment alongside a demanding curriculum may reduce students' satisfaction, particularly with study conditions and their coping with study load.

**Figure 1.**
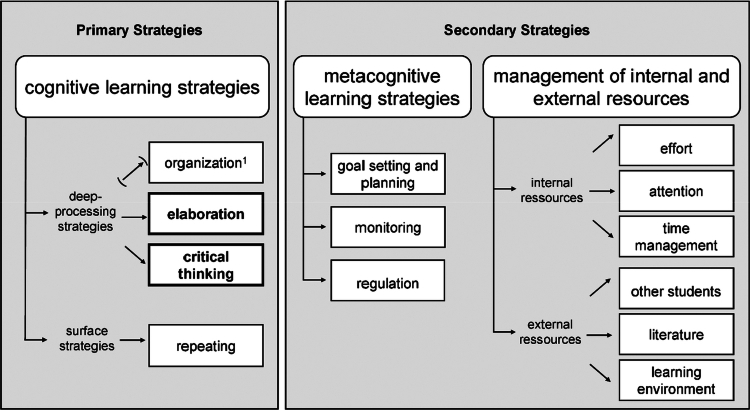
*Overview of the classification of learning strategies according to Wild and colleagues* [29,30]*, figure adapted from Wild* [29]. ^1^This study focused on deep-processing strategies, specifically elaboration and critical thinking. For completeness, it is acknowledged that organisational strategies are also sometimes classified as deep-processing strategies, although they were not included in the present analysis.

Conversely, prior professional experience may enhance satisfaction with the study contents, as students tend to report higher satisfaction when they perceive the curriculum as practically and professionally relevant [24,25]. Students with vocational training in the medical field or a prior academic degree may possess more extensive practical or academic prior knowledge. In this context, deep-processing strategies such as elaboration and critical thinking may play a different role for students with prior professional or academic experience than for students without such backgrounds. In self-regulated learning research, elaboration refers to linking new information with existing knowledge structures [26,27], whereas critical thinking involves actively evaluating and questioning learning content [28]. According to the model by Wild and colleagues [28–30], deep-processing strategies such as elaboration and critical thinking are part of cognitive learning strategies (see Figure 1) and should be preferred over surface strategies [28].

Both strategies may be especially relevant in the context of prior professional or academic experience, as students with pre-medical qualifications may be able to connect new learning content more closely to existing practical or academic experiences. Specifically, deep-processing strategies may contribute more strongly to satisfaction with study contents when students are able to relate learning material to prior professional or academic experiences. Prior knowledge is a key component of self-regulated learning and plays an important role in learners' use of self-regulated learning strategies [31–34]. Moreover, previous studies indicate positive associations between self-regulated learning strategies and study satisfaction [34–36]. We therefore examined whether pre-medical qualifications moderate the association between deep-processing strategies and satisfaction with study contents and hypothesised that these associations would be stronger among students with vocational training or prior academic degrees.

Using Germany as a case example, where practical pre-qualifications play an important role in medical school admission, this study contributes to research on the diversification of medical education. This study aims to analyse the associations between pre-qualifications and study satisfaction, and to investigate the role of part-time employment and deep-processing learning strategies in this relationship. As vocational training in the medical field and an academic degree constitute longer and more substantial pre-qualifications, these form the primary focus of our analysis on study satisfaction, while voluntary service is examined exploratorily. The analyses further account for relevant control variables such as age, gender, undergraduate GPA, semester and medical school. Based on our considerations, the following hypotheses and research questions are investigated.

1. Moderation hypotheses

The association between deep-processing learning strategies (elaboration and critical thinking) and satisfaction with study contents is stronger among students

a) with completed vocational training in the medical field and

b) with a prior academic degree.

2. Mediation hypotheses

Students with

a) completed vocational training and

b) a prior academic degreeare more likely to finance their studies through part-time employment, which is associated with lower satisfaction with study conditions and coping with study load.

3. Exploratory research question: How do medical students with completed voluntary service differ in their study satisfaction?

## Methods

### Participants and design

We conducted a cross-sectional, multicenter study at five public medical schools in south-west Germany (the federal state of Baden-Württemberg) between the German winter semester 2019/2020 and summer semester 2021. All five medical schools prepare students for the same nationally standardised state examinations and base their curricula on the National Competence-Based Learning Objectives Catalogue for Medicine (NKLM), which promotes consistency and comparability across German medical schools [37]. Recruitment of medical students (3^rd^, 6^th^, 10^th^ semester, final year) was via email or during lectures. Medical students gave their written informed consent before the start of the study and their participation was anonymous and voluntary. The authors distributed book vouchers as an incentive for participation.

### Measures

This study is a sub-analysis of the collaborative research project ‘Pre-qualifications for human medicine’, which examines medical students with completed pre-qualifications before beginning their medical studies. Three delineated sub-analyses have already emerged from this dataset, namely the relationship between prior qualifications and academic success [18], burnout [38], and specialist training [39]. The overall questionnaire assessed medical students' socio-demographic background and medical school-related questions such as current semester and undergraduate grade point average (GPA; in German ‘Abitur’, with lower numbers indicating better grades). Students were asked whether they finance their studies (in part) through part-time employment. Furthermore, students reported their *professional and academic pre-qualifications,* which were operationalized as the following dichotomous variables:

(i) Completed *vocational training in the medical field*
(ii) *Academic degree*, Bachelor's or Master's degree
(iii) Completed *voluntary service* of at least 11 months

These variables were selected because they represent distinct forms of pre-qualifications considered within the quota-based German medical school admission system [10,40]. In particular, vocational training in the medical field and voluntary service are relevant criteria within the Additional Aptitude Quota (‘Zusätzliche Eignungsquote’, ZEQ) and the University Selection Procedure (‘Auswahlverfahren der Hochschulen’, AdH), whereas applicants with prior academic degree are admitted through a separate quota for second-degree applicants [10,18,40].

### Study satisfaction

Study satisfaction was assessed by the Study Satisfaction Questionnaire—Short Form (FB-SZ-K; [8,41]. Students rate their satisfaction on a graphical scale from 0-100. The questionnaire consists of nine items, which are evenly allocated to three scales (see Table A1 in the appendix for an overview of all items). Cronbach's *α* is reported from Westermann et al. [41].

- *Satisfaction with the study contents*, *α* = .87
- *Satisfaction with the study conditions*, *α* = .71
- *Satisfaction with coping with study load*, *α* = .71

### Learning strategies

Deep-processing learning was assessed by using two scales *elaboration* (Cronbach's *α* = .65–.72) and *critical thinking* (Cronbach's *α* = .73–.79) from the Learning Strategies of University Students questionnaire - short version (LIST-K [42]. Medical students rated their strategy use on a five-point-scale from 1 (rarely) to 5 (often). All items (three per strategy) are provided in Table A1 in the appendix.

### Statistical analysis

We performed the statistical analysis using R 4.5.1. The level of significance was set at *α* = .05. Descriptive information on specific vocational training categories and prior academic degrees is provided in the sample description. For the main analyses, however, these pre-qualifications were operationalized as broader dichotomous variables in line with their role within the German medical school admission system [10,18,40].

To test the hypotheses and the explorative research question, we calculated a structural equation model (SEM) with prior confirmatory factor analysis (CFA) using the lavaan package [43]. In the first step we calculated the CFA including all latent variables. The latent variables were the three satisfaction scales and the learning strategies elaboration and critical thinking. In preparation for the CFA, the satisfaction scales were brought to a comparable value range as the learning strategy scales (0-10 instead of 0-100), in order to avoid instabilities in the estimation. Model fit was evaluated based on common recommendations [44].

In the second step we added the manifest variables and calculated the SEM. The manifest variables were the moderator variables vocational training in the medical field (0 = no, 1 = yes) and academic degree (0 = no, 1 = yes). Further manifest variables were voluntary service (0 = no, 1 = yes), the mediator variable *part-time employment* to finance studies (0 = no, 1 = yes) and the control variables age (mean-centred), undergraduate GPA (mean-centred) and gender (0 = female, 1 = male). Medical school (A, B, C, D, ref: E) and semester (3^rd^, 6^th^, 10^th^, ref: final year) were entered as dummy-coded variables. Medical schools were included as pseudonymized control variables (A-E) to account for institutional differences in study satisfaction while ensuring institutional anonymity.

**Figure 2.**
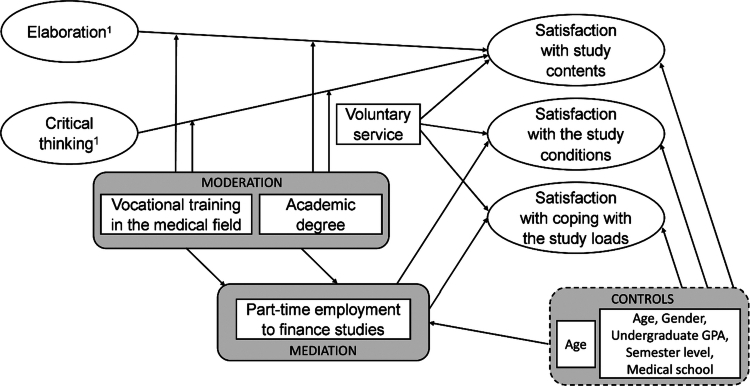
*Overview on all latent (ellipses) and manifest (rectangles) variables in the structural model.* ^1^Factor scores were estimated from the CFA and were entered as manifest variables in the model.

To build interaction terms with the moderator variables, factor scores for elaboration and critical thinking were estimated and extracted from the CFA. Factor scores were z-standardised before building interaction terms. Although this enabled us to model the interactions, it should be noted that factor scores are only estimates of latent variables and do not explicitly account for measurement errors. All dichotomous variables were modelled metrically. Figure 2 provides an overview of all variables in the structural model. Standardised regression coefficients were calculated to determine the strength of the associations between the variables, indicating how much the dependent variable changes (in SD units) when the predictor increases by one SD unit.

## Results

### Sample description

The overall sample size was *N* = 2,370 out of approximately 5,094 invited students, corresponding to an overall response rate of 46.5%. Participating medical students were in their 3^rd^ (*n* = 635, 26.8%), 6^th^ (*n* = 729, 30.8%) and 10^th^ semester (*n* = 485, 20.5%) or in their final year (*n* = 521, 22.0%). More than half of the students were female (*n* = 1,521, 64.7%). Of all medical students, *n* = 655 (27.6%) had completed vocational training in the medical field (vs. *n* = 1,707, 72.3% had not). Among these students, the most common vocational backgrounds were paramedics (*n* = 283, 43.2%) and nurses (*n* = 238, 36.3%), followed by professions in the broader health sector (*n* = 128, 19.5%; including speech and language therapists, physiotherapists, and medical assistants). A small number of students reported vocational training in other areas such as the pharmaceutical sector or geriatric care (*n* = 6, 0.9%).

A total of *n* = 145, 6.1% medical students stated that they had completed another academic degree prior to starting their studies (vs. *n* = 2,214, 93.9% had not). The most frequently reported prior degrees were in STEM subjects (*n* = 44, 30.3%; including mathematics, computing, biology, chemistry, and physics) and in the medical field (*n* = 41, 28.3%; including dentistry, molecular medicine, and human biology). Other prior degrees were reported in psychology (*n* = 14, 9.7%) and various additional fields (*n* = 46, 31.7%), including nursing and health sciences, pedagogy and educational sciences, home economics, and nutritional sciences.

A total of *n* = 546 (23.1%) students stated that they had completed voluntary service before starting their studies (vs. *n* = 1,813, 76.9% had not). Voluntary service was most commonly completed in health care settings (*n* = 215, 39.5%), followed by child and youth welfare (*n* = 55, 10.1%), care for people with disabilities (*n* = 28, 5.1%), and elderly care (*n* = 16, 2.9%). Overall, *n* = 230 (42.3%) students reported voluntary service in ‘other’ fields, that were not further specified. Overall *n* = 1,490 (62.9%) medical students indicated part-time employment to finance their studies. Please refer to Schröpel et al [18]. for a detailed description of the sample. Table 1 provides an overview on the descriptive statistics of the learning strategies and satisfaction scales.

**Table 1.** Descriptive statistics including Pearson's correlation coefficients between all metric study variables.

		1	2	3	4	5
1	Elaboration					
2	Critical Thinking	.40				
3	Satisfaction with the study contents	.19	.06			
4	Satisfaction with the study conditions	.03	−.04	.38		
5	satisfaction with coping with study load	.09	.07	.36	.44	
	*n*	2338	2330	2353	2347	2356
	*M*(*SD)*	3.48(0.77)	2.98(0.84)	8.63(1.68)	4.91(2.72)	6.3(2.65)
	range	1–5	1–5	0–10	0–10	0–10
	Skewness	−0.23	0.06	−1.46	−0.03	−0.49
	Kurtosis	−0.28	−0.27	2.64	−1.07	−0.73
	Cronbach's α	.56	.76	.88	.87	.89

### Results of the structural equation model

Before running the analysis, we tested relevant assumptions. To account for missing values in our data (<2%), we used the full information maximum likelihood (FIML) approach. Overall, all assumptions were met. However, as we detected multivariate outliers in our data and multivariate normality was not met, we used robust maximum likelihood estimation (MLR) for the CFA to calculate an unbiased measurement model. For the SEM, we used maximum likelihood estimation (ML) in combination with bootstrapping (2000 samples). Significance decisions in the SEM were based on the 95% bias-corrected and accelerated Bootstrap (BCa) confidence intervals (CI). An effect was considered significant if the CI did not include zero.

The results of the CFA overall showed a good fit between the empirical data and the measurement model, χ^2^(80) = 914.613, *p* < .001, CFI = .95, TLI = .93, RMSEA = .06, 90% CI [.06, .07], SRMR = .05. The model showed an AIC of 125570.31 and a BIC of 125887.63. All standardised factor loadings were significant and between 0.50 and 0.92, see Table A1 in the Appendix.

In the second step we calculated the SEM, see Table 2 for all results. Overall, the model showed a good model fit. The *R* ^2^ for ‘satisfaction with the study contents’ was 0.15. Higher satisfaction was associated with a more frequent use of 'elaboration' (ß = 0.51) and a less frequent use of strategy ‘critical thinking’ (ß = −0.31), as well as a lower age (ß = −0.09). The medical school also affected satisfaction levels (A, C, D > E). However, we were unable to identify any moderating effects or associations with vocational training, a prior academic degree, voluntary service, gender, undergraduate GPA and semester level.

The variance in ‘part-time employment to finance studies’ (*R* ^2^ = .04) could not be explained by age. However, medical students with vocational training (ß = 0.18) and medical students with academic degree (ß = 0.05) more often indicated to finance studies through part-time employment than medical students without training or without degree.

We identified an *R* ^2^ of 0.10 for ‘satisfaction with the study conditions’. Medical students financing studies through part-time employment (ß = −0.07), and older students (ß = −0.12) were less satisfied. However, 3^rd^ semester students (ß = 0.12) reported higher satisfaction (compared to final year students) and the medical school also had an effect on satisfaction levels (A, B, C, D > E). No effects were observed for the variables vocational training, academic degree, voluntary service and undergraduate GPA.

The *R* ^2^ for ‘satisfaction with coping with study load’ was 0.07. Students financing studies through part-time employment (ß = 0.08), and male students (ß = 0.09) reported higher levels of satisfaction with coping with study load, whereas older students (ß = −0.18), students with completed voluntary service (ß = −0.06), and 3^rd^ (ß = −0.27) and 6^th^ semester students (ß = −0.15) (compared to final year students) reported lower levels. There were no effects of vocational training, academic degree, medical school, and undergraduate GPA.

**Figure 3.**
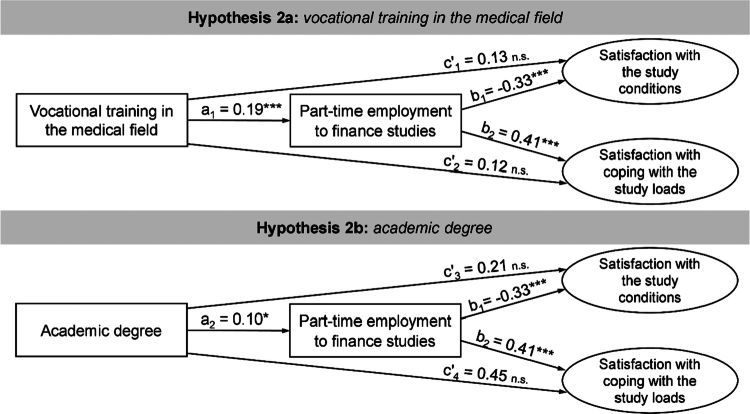
*Illustration of the coefficients (unstandardised) of the mediation analysis with mediation paths a and b and the direct effect c'*. *Note*: Latent variables (ellipses) and manifest variables (rectangles) *p* < .05*, *p* < .01**, *p* < .001***.

Financing studies through part-time employment fully mediated the relationship between vocational training in the medical field and satisfaction with the study conditions, ab_1_ = −0.06, 95% CI [−0.10, −0.03], ß = −0.01, and between vocational training and satisfaction with coping with study load, ab_2_ = 0.08, 95% CI [0.04, 0.13], ß = 0.01. Also, part-time employment fully mediated the relationship between academic degree and satisfaction with the study conditions, ab_3_ = −0.03, 95% CI [−0.08, −0.01], ß = −0.003, and between academic degree and satisfaction with coping with study load, ab_4_ = 0.04, 95% CI [0.01, 0.09], ß = 0.004. Figure 3 illustrates the effects of the mediation analyses.

**Table 2.** Results of regression paths between the study variables, significant effects in bold.

	b	SE	p	95% CI	ß
**Satisfaction with the study contents ~**					
elaboration	**0.85**	**0.06**	**<.001**	**[0.73, 0.98]**	**0.51**
critical thinking	**−0.52**	**0.06**	**<.001**	**[** **−0.65, −0.4]**	**−0.31**
vocational training in the medical field	−0.11	0.11	.325	[−0.33, 0.11]	−0.03
academic degree	0.12	0.17	.504	[−0.21, 0.48]	0.02
elaboration*vocational training	−0.06	0.13	.633	[−0.30, 0.19]	−0.02
critical thinking*vocational training	0.08	0.12	.522	[−0.16, 0.3]	0.02
elaboration*academic degree	0.41	0.36	.251	[−0.20, 1.21]	0.06
critical thinking*academic degree	−0.24	0.34	.478	[−0.996, 0.36]	−0.04
age	**−0.04**	**0.02**	**.020**	**[** **−0.08, −0.01]**	**−0.09**
male gender	0.03	0.07	.728	[−0.13, 0.17]	0.01
undergraduate GPA	0.14	0.1	.155	[−0.05, 0.33]	0.04
voluntary service	−0.14	0.08	.088	[−0.31, 0.02]	−0.04
medical school A	**0.46**	**0.10**	**<.001**	**[0.25, 0.66]**	**0.13**
medical school B	0.18	0.20	.368	[−0.22, 0.55]	0.02
medical school C	**0.43**	**0.10**	**<.001**	**[0.22, 0.62]**	**0.12**
medical school D	**0.40**	**0.14**	**.005**	**[0.11, 0.67]**	**0.07**
3^rd^ semester	0.17	0.14	.218	[−0.10, 0.43]	0.04
6^th^ semester	0.09	0.12	.439	[−0.13, 0.33]	0.02
10^th^ semester	0.16	0.11	.141	[−0.05, 0.38]	0.04
**part-time employment to finance studies ~**					
vocational training in the medical field	**0.19**	**0.02**	**<.001**	**[0.14, 0.24]**	**0.18**
academic degree	**0.10**	**0.04**	**.020**	**[0.01, 0.17]**	**0.05**
age	0.01	0.003	.145	[−0.002, 0.01]	0.04
**satisfaction with the study conditions ~**					
financing studies by own earnings	**−0.33**	**0.10**	**<.001**	**[** **−0.52, −0.13]**	**−0.07**
vocational training in the medical field	0.13	0.16	.426	[−0.19, 0.44]	0.02
academic degree	0.21	0.25	.406	[−0.30, 0.69]	0.02
age	**−0.08**	**0.03**	**<.001**	**[** **−0.13, −0.03]**	**−0.12**
male gender	0.02	0.11	.884	[−0.18, 0.25]	0.003
undergraduate GPA	−0.07	0.15	.623	[−0.36, 0.21]	−0.02
voluntary service	−0.16	0.12	.183	[−0.42, 0.07]	−0.03
medical school A	**1.23**	**0.14**	**<.001**	**[0.95, 1.50]**	**0.25**
medical school B	**1.27**	**0.26**	**<.001**	**[0.76, 1.76]**	**0.11**
medical school C	**0.49**	**0.14**	**<.001**	**[0.2, 0.75]**	**0.10**
medical school D	**0.57**	**0.20**	**.005**	**[0.16, 0.99]**	**0.07**
3^rd^ semester	**0.63**	**0.19**	**.001**	**[0.28, 1.01]**	**0.12**
6^th^ semester	−0.20	0.16	.204	[−0.51, 0.12]	−0.04
10^th^ semester	−0.11	0.15	.471	[−0.42, 0.20]	−0.02
**satisfaction with coping with study load ~**					
financing studies by own earnings	**0.41**	**0.11**	**<.001**	**[0.21, 0.63]**	**0.08**
vocational training in the medical field	0.12	0.18	.493	[−0.21, 0.50]	0.02
academic degree	0.45	0.28	.103	[−0.09, 0.99]	0.04
age	**−0.12**	**0.03**	**<.001**	**[** **−0.18, −0.07]**	**−0.18**
male gender	**0.49**	**0.12**	**<.001**	**[0.24, 0.72]**	**0.09**
undergraduate GPA	0.16	0.17	.353	[−0.18, 0.49]	0.03
voluntary service	**−0.36**	**0.14**	**.013**	**[** **−0.64, −0.08]**	**−0.06**
medical school A	0.30	0.16	.067	[−0.02, 0.62]	0.05
medical school B	0.07	0.31	.821	[−0.50, 0.73]	0.01
medical school C	0.24	0.16	.129	[−0.07, 0.56]	0.04
medical school D	−0.35	0.24	.141	[−0.83, 0.11]	−0.04
3^rd^ semester	**−1.60**	**0.22**	**<.001**	**[** **−2.05, −1.19]**	**−0.27**
6^th^ semester	**−0.85**	**0.18**	**<.001**	**[** **−1.22, −0.50]**	**−0.15**
10^th^ semester	−0.05	0.18	.793	[−0.37, 0.32]	−0.01

Χ^2^(173) = 1021.041, *p* < .001, CFI = .94, TLI = .92, RMSEA = .05, 90% CI [0.04, 0.05], SRMR = 0.03, AIC = 85641.07, BIC = 86110.44. Note. b = unstandardised regression coefficient; SE = standard error; β = standardised regression coefficient (SD units); CI = confidence interval. 95% bootstrap confidence intervals are reported. Dichotomous variables were coded as 0 = no and 1 = yes, except gender (0 = female, 1 = male). Medical school (reference: medical school E) and semester (reference: final year) were dummy-coded.

## Discussion

This multicenter, cross-sectional study of medical students in different semesters aimed to analyse differences in study satisfaction (i.e., satisfaction with the study contents, with the study conditions and with the coping with study load) between students with and without professional and academic pre-qualifications (i.e., vocational training in the medical field, academic degree, voluntary service). In this context, a structural equation modelling (SEM) approach was employed to examine the role of the learning strategies elaboration and critical thinking, and the financing of studies through part-time employment while accounting for relevant control variables.

### Deep-processing learning, pre-qualifications and satisfaction with the study contents

The moderation hypotheses could not be confirmed. The strength of the association between ‘elaboration’ or ‘critical thinking’, and ‘satisfaction with the contents of studies’ did not differ between medical students with completed vocational training in the medical field and students without (Moderation Hypothesis 1a), nor between students with a prior academic degree and students without (Moderation Hypothesis 1b).

We expected a stronger link between the deep-processing strategies and satisfaction levels among pre-qualified students, as previous research indicates that citing professional and content-related reasons for choosing a field of study [25], having a personal interest in the subject, and perceiving the curriculum as vocationally relevant [24] are positively related to satisfaction with the study contents. Regardless of pre-qualification status, elaboration was positively, and critical thinking negatively, associated with study satisfaction. Notably, these associations were among the strongest effects observed in the model. Compared with the generally small effects of demographic characteristics and pre-qualifications, deep-processing learning strategies showed comparatively substantial associations with satisfaction with the study contents.

This finding suggests that students who more frequently connect new material to prior knowledge also tend to report higher satisfaction with the study contents. Although causal conclusions cannot be drawn from the cross-sectional design, the comparatively strong association highlights elaboration as a potentially relevant factor in students' learning experiences. Accordingly, teaching approaches that encourage students to actively connect new content with existing knowledge and experiences may support elaborative learning. In a qualitative study, medical students described peer explanations, case discussions, and encounters with patients as learning experiences that enhanced their use of elaboration strategies [27]. Similarly, reverse causality cannot be ruled out, as students with higher satisfaction may be more likely to engage in elaboration as a learning strategy.

While the positive association between elaboration and satisfaction levels aligns with previous findings on SRL strategies, the negative association of critical thinking stands in contrast to this literature [35,36]. Although critical thinking as a deep-processing learning strategy can foster a deeper understanding of course content, students reporting more frequent use of critical thinking also reported lower satisfaction with study contents. Several explanations may account for this finding. Students who engage more strongly in critical thinking may hold higher expectations regarding the quality, relevance, or coherence of the curriculum and may therefore evaluate study contents more critically. Similarly, critical thinking may be associated with a greater awareness of curricular limitations or inconsistencies, resulting in lower satisfaction ratings despite high academic engagement. At the same time, reverse causality cannot be ruled out. Students who are less satisfied with their studies may be more likely to question and critically evaluate curricular content. Given the cross-sectional design, the direction of the observed association remains unclear and should be investigated in future longitudinal research. Nevertheless, critical thinking should not be viewed as negatively. Learning-strategy research consistently highlights its importance for deeper learning and academic development [28]. Actively questioning and discussing course content remains essential for learning and should be encouraged.

A study with a similar focus on elaboration and critical thinking did not find a direct connection between deep-processing strategies and satisfaction with the study content; instead, deep-processing strategies influenced satisfaction indirectly through increased self-efficacy [45]. In that study, however, elaboration and critical thinking were merged into a single scale, which may have neutralised opposing effects of the two strategies. This interpretation remains speculative, as the studies differ methodologically and consider different variables. Given that other research also points to a connection between SRL and self-efficacy beliefs [46,47], future studies should examine the role of self-efficacy in the relationship between deep-processing learning and satisfaction.

Our findings suggest that different deep-processing learning strategies may relate to study satisfaction in different ways. Nonetheless, medical students and other higher-education students must regulate their learning effectively to meet substantial academic demands [45,48–50]. Given that SRL is related to academic achievement [31,51–53], it remains a valuable competence that should be actively supported in medical education. Further research is needed to examine its effect on study satisfaction.

### Financing studies through part-time employment as mediating variable

The mediation hypotheses were partly confirmed. Medical students who had completed vocational training (Mediation Hypothesis 2a) or had a prior academic degree (Mediation Hypothesis 2b) financed their studies more often through part-time employment than students without these qualifications, and were less satisfied with the conditions of their studies. However, contrary to our hypothesis, they were more satisfied with coping with study load. This finding suggests that employment may not be uniformly associated with negative study experiences and that its role may depend on broader individual and contextual factors.

Studying at a public university in Germany is tuition-free and funded by taxes [54], yet students must still cover their living expenses. Parental support is the primary source of student funding in Germany [55], but for medical students who have already completed vocational training or a degree, full financial support from parents may no longer be feasible. As a result, students may rely on part-time employment to finance their studies and living expenses. The association between vocational training and part-time employment was small to moderate, whereas the corresponding association for prior academic degree was very small. This pattern suggests that vocational training may be more strongly associated with students' likelihood of financing their studies through employment than prior academic degree.

Medical studies are highly demanding, combining traditional lectures with clinically responsive activities [56,57]. The dense schedule could make it difficult to maintain part-time work, potentially leading to frustration. However, the association between part-time employment and satisfaction with study conditions was small, suggesting that employment alongside medical studies may be associated with students' perceptions of study conditions only to a limited extent. At the same time, the positive association between part-time employment and satisfaction with coping with study load was small. One possible explanation is that students who work alongside their studies may experience additional structure, financial security, or opportunities to develop professional competencies, which could be associated with slightly higher satisfaction with coping with study load. However, given the small effect size and the cross-sectional design, alternative explanations are equally plausible. For example, students who choose to work alongside their studies may differ from other students in ways that also influence their satisfaction with coping with study load. Other studies suggest that employment alongside studying does not necessarily have a negative impact on study satisfaction, but rather that its effects depend on the extent of working hours [58]. Although the associations involving part-time employment were small, they suggest that the needs of medical students who work alongside their studies should be considered when designing curricular structures. Examples may include asynchronous learning formats and predictable scheduling that facilitate the planning of part-time employment alongside the demanding requirements of medical training.

Part-time employment statistically mediated the associations between pre-qualifications and study satisfaction. However, the indirect effects were very small, and the explained variance of the mediator was low, suggesting limited practical significance despite statistical significance. Given the cross-sectional design and the relatively small number of students with a prior academic degree, these findings should be interpreted cautiously. Rather than demonstrating a causal mechanism, the results indicate that part-time employment may represent one possible pathway linking pre-qualifications and study satisfaction. Future research should investigate additional factors that may contribute to these associations.

### Other associations with study satisfaction

In an exploratory manner, we examined the associations between voluntary service and the three satisfaction dimensions. Completion of voluntary service was associated with slightly lower satisfaction with coping with study load. Given the small effect size, this finding should be interpreted cautiously. One possible explanation is that students who previously engaged in practical and socially meaningful activities may perceive the transition to a highly theoretical and performance-oriented learning environment as more demanding.

Among our control variables, we identified additional influencing factors. Differences between medical schools were observed for satisfaction with study contents and study conditions, whereas no differences emerged for coping with study load. As medical school was included as a control variable rather than a primary focus of the study, these findings should be interpreted cautiously. Older age was associated with lower satisfaction across all three dimensions, possibly reflecting changing life circumstances. Regarding semester level, third-semester students reported higher satisfaction with study conditions than final-year students, consistent with evidence of inconsistent supervision and a perceived lack of adequate preparation for clinical practice in the final year [59]. Conversely, third- and sixth-semester students reported lower satisfaction with coping with study load than final-year students. One possible explanation is that students in later stages of training may feel more familiar with the demands of medical school; however, the underlying reasons for this difference remain unclear. Overall, men reported higher satisfaction with coping with study load, consistent with literature on burnout among medical students [60]. With the exception of semester-related differences in coping with study load, most associations observed for voluntary service and demographic variables were small in magnitude, indicating that unmeasured factors may also play a substantial role, such as teaching variables [61–63]. Their practical significance should therefore be interpreted cautiously despite statistical significance.

### Strengths and limitations

A particular strength of our study is that it is a large, multicenter study conducted at five public medical schools in Germany, at different semester levels. We chose an SEM approach, which enabled us to simultaneously estimate several relationships between latent and manifest variables while controlling for measurement errors. However, due to the cross-sectional design, no causal conclusions can be drawn. The internal consistency of the variable elaboration was low, which limits the interpretation of the results. Another limitation is that the study was conducted during the COVID-19 pandemic, which may have influenced study satisfaction and thus limits the generalizability of the findings. In addition, although pre-qualifications may overlap within individuals, vocational training, academic degree, and voluntary service were operationalized as separate dichotomous variables. These categories were deliberately selected because they reflect distinct admission-relevant pre-qualifications in the German medical school admission system. Accordingly, their associations with other variables should be interpreted as independent statistical effects. However, the analyses do not allow conclusions about specific vocational, academic, or voluntary-service backgrounds. The measurement of part-time employment was limited to a dichotomous indicator and therefore could not capture important aspects of students' employment situations, such as weekly working hours, the type of employment (e.g., clinical versus non-clinical work), the extent of financial reliance on employment income, or the perceived burden associated with working alongside studying. As these factors may influence study satisfaction in different ways, the mediation findings should be interpreted with caution. More detailed measures of employment and financial circumstances should be included in future research. Furthermore, additional factors that were not assessed in the present study may contribute to study satisfaction, including students' socioeconomic background, and financial support structures.

### Conclusion

Relating new learning content to prior knowledge and experience was positively associated with higher satisfaction with study contents, with no evidence of differences in this association between students who had completed vocational training and those who held a prior academic degree. Medical schools may consider creating opportunities for students to connect theoretical knowledge with prior experiences and real-world applications, as such approaches could support elaborative learning. In contrast, more frequent use of critical thinking was associated with lower satisfaction with study contents, highlighting the need for further research to better understand this relationship and its underlying mechanisms. Although part-time employment was associated with lower satisfaction with study conditions, it was also associated with slightly higher satisfaction with coping with study load. Given the small effect sizes, these findings should be interpreted cautiously and may indicate that employment has both potential challenges and potential benefits for students. Although the study was conducted within the German medical education system, the findings may inform discussions about supporting increasingly diverse student populations. Across many countries, medical schools have sought to broaden access for applicants from underrepresented and non-traditional backgrounds through widening participation initiatives [14–17]. In a broader sense, the targeted admission of applicants with prior professional or academic experience may also be viewed as these efforts [20]. Our study therefore contributes to a better understanding of students with professional or academic pre-qualifications, a group that has received comparatively little attention in research on study satisfaction, and may help inform admission policies, student support strategies, and widening participation initiatives aimed at fostering a more inclusive medical workforce.

## Data Availability

The datasets presented in this article are not readily available because of the high confidentiality of the data. The authors of the study received permission from the Medical Faculty of Tübingen to conduct the study and to collect these data only if they were not made publicly available without individual permission for specific questions (i.e., on request). Requests to access the datasets should be directed to psychosomatik@med.uni-tuebingen.de.

## Previous presentation

Conference presentation: GMA - Gesellschaft für medizinische Ausbildung, Düsseldorf, Germany (2025, 08.-10. August). The presentation was in German language and is not publicly available.

## Appendix

**Table A1.** (Standardised) factor loadings and explained variance for the items of the latent variables in the measurement model.

Factor	Items (translated from German)	Factor loadings	*SE*	Standardised factor loadings	*p*	*R* ^2^
Satisfaction with the study contents	I really enjoy what I am studying	1.74	0.04	0.92	<.001	0.85
	Overall, I am happy with my current studies	1.70	0.05	0.83	<.001	0.69
	I find my studies really interesting	1.30	0.05	0.80	<.001	0.64
Satisfaction with the study conditions	The external circumstances in which my subject is studied are frustrating	2.34	0.05	0.76	<.001	0.58
	I wish the study conditions at the university were better	2.80	0.04	0.90	<.001	0.81
	Too little attention is paid to students' concerns at my university	2.50	0.04	0.84	<.001	0.70
Satisfaction with coping with study load	My studies are consuming me	2.63	0.05	0.90	<.001	0.81
	I find it difficult to combine my studies with other commitments	2.20	0.05	0.76	<.001	0.57
	I often feel tired and exhausted because of my studies	2.66	0.04	0.89	<.001	0.79
Elaboration	I try to relate new concepts or theories to concepts and theories I am already familiar with	0.54	0.03	0.57	<.001	0.32
	I think of specific examples for certain learning strategies	0.58	0.03	0.50	<.001	0.25
	I relate what I have learned to my own experience	0.60	0.03	0.58	<.001	0.34
Critical Thinking	I ask myself if the text I'm currently working on is really convincing enough	0.66	0.02	0.63	<.001	0.40
	I tend to take a critical approach to most texts	0.83	0.02	0.81	<.001	0.65
	I also think critically about what I have learned	0.73	0.02	0.72	<.001	0.51

## References

[1] Slavin S . Reimagining well-being initiatives in medical education: shifting from promoting wellness to increasing satisfaction. Acad Med. 2021;96(5):632–4. doi: 10.1097/ACM.0000000000004023 33635840

[2] Lehnchen J , Helmer SM , Heinrichs K , et al. Assessment of study conditions, needs for action and students’ mental health in Germany–results from the cross-sectional StudiBiFra study. J Public Health (Bangkok). 2025;1–11. doi: 10.1007/s10389-024-02387-9

[3] An M , Ma X , Wu H . Medical students' academic satisfaction: social cognitive factors matter. Med Educ. 2023;57(12):1239–47. doi: 10.1111/medu.15070 36868559

[4] Grebennikov L , Shah M . Monitoring trends in student satisfaction. Tertiary Education and Management. 2013;19(4):301–22. doi: 10.1080/13583883.2013.804114

[5] Santini FdO , Ladeira WJ , Sampaio CH , et al. Student satisfaction in higher education: a meta-analytic study. Journal of Marketing for Higher Education. 2017;27(1):1–18. doi: 10.1080/08841241.2017.1311980

[6] Kuensting J , Lipowsky F . Motivation for choosing a teacher education program and personality traits as predictors for study satisfaction and strategy use. Zeitschrift für Pädagogische Psychologie. 2011;25(2):105–14. doi: 10.1024/1010-0652/a000038

[7] Rahmatpour P , Nia HS , Peyrovi H . Evaluation of psychometric properties of scales measuring student academic satisfaction: a systematic review. J Educ Health Promot. 2019;8(1):256. doi: 10.4103/jehp.jehp_466_19 32002428 PMC6967218

[8] Westermann R , Elke H , Spies K , et al. Identifikation und Erfassung von Komponenten der Studienzufriedenheit. Psychologie in Erziehung und Unterricht. 1996.

[9] Apenburg E . Untersuchungen zur Studienzufriedenheit in der heutigen Massenuniversität: Peter Lang Gmbh. Internationaler Verlag Der Wissenschaften; 1980.

[10] Erschens R , Herrmann-Werner A , Schaffland TF , et al. Association of professional pre-qualifications, study success in medical school and the eligibility for becoming a physician: a scoping review. PLoS One. 2021;16(11):e0258941. doi: 10.1371/journal.pone.0258941 34762678 PMC8584759

[11] Oyoun Alsoud L , West K , Sorrell S , et al. A cross-sectional study of newly established medical schools in the United States: student body diversity remains an unmet challenge. Med Educ Online. 2025;30(1):2487660. doi: 10.1080/10872981.2025.2487660 40176252 PMC11966980

[12] Gishen F , Lokugamage A . Diversifying the medical curriculum. BMJ. 2019;364:l300. doi: 10.1136/bmj.l300 30674468

[13] Fan EY , Megafu O , Lee J , et al. Diversifying the surgical workforce: understanding barriers to inform solutions. J Surg Educ. 2025;82(3):103418. doi: 10.1016/j.jsurg.2024.103418 39818081

[14] Ravulapalli KC , Arroyave Caicedo NM , Zahra D , et al. Quantitative analysis of challenges encountered by UK widening participation medical students in comparison with their non-widening participation peers. J Med Educ Curric Dev. 2024;11. doi: 10.1177/23821205241249012 PMC11131392 38808124

[15] Groene OR , Huelmann T , Hampe W , et al. German physicians and medical students do not represent the population they serve. Healthcare (Basel). 2023;11(12):1662. doi: 10.3390/healthcare11121662 37372780 PMC10298415

[16] Rees E , Woolf K . Selection in context: the importance of clarity, transparency and evidence in achieving widening participation goals. Med Educ. 2020;54(1):8–10. doi: 10.1111/medu.14023 31849095

[17] Wouters A . Getting to know our non-traditional and rejected medical school applicants. Perspect Med Educ. 2020;9(3):132–4. doi: 10.1007/S40037-020-00579-Z 32270368 PMC7283413

[18] Schröpel C , Festl-Wietek T , Herrmann-Werner A , et al. How professional and academic pre-qualifications relate to success in medical education: results of a multicentre study in Germany. PLoS One. 2024;19(3):e0296982. doi: 10.1371/journal.pone.0296982 38457481 PMC10923489

[19] Amelung D , Zegota S , Espe L , et al. Considering vocational training as selection criterion for medical students: evidence for predictive validity. Adv Health Sci Educ Theory Pract. 2022;27(4):933–48. doi: 10.1007/s10459-022-10120-y 35794434 PMC9606097

[20] Müller-Hilke B , Finger C , Hampe W . Neues Zulassungsverfahren Humanmedizin: höhere individuelle Gerechtigkeit, aber Verstärkung des Landarztmangels? Bundesgesundheitsblatt-Gesundheitsforschung-Gesundheitsschutz. 2024;67(2):225–232. doi: 10.1007/s00103-023-03825-x 38197927 PMC10834559

[21] Tsikas SA , Kieca M . Student background, admission routes, and academic success: a structural mediation analysis. BMC Med Educ. 2026;26(1):578. doi: 10.1186/s12909-026-09068-z 41872821 PMC13064409

[22] Nidogon Višnjić S , Pažur Aničić K , Divjak B . A systematic review of the literature on student work and academic performance. Industry and Higher Education. 2024;38(5):473–84. doi: 10.1177/09504222241241974

[23] Brandstätter H , Farthofer A . Influence of part-time work on university students' academic performance. Zeitschrift für Arbeits-und Organisationspsychologie. 2003;47(3):134–45.

[24] Schiefele U , Jacob-Ebbinghaus L . Lernermerkmale und Lehrqualität als bedingungen der studienzufriedenheit. Zeitschrift für pädagogische Psychologie. 2006;20(3):199–212. doi: 10.1024/1010-0652.20.3.199

[25] Hiemisch A , Westermann R , Michael A . Die Abhängigkeit der Zufriedenheit mit dem Medizinstudium von Studienzielen und ihrer Realisierbarkeit. Zeitschrift für Psychologie/Journal of Psychology. 2005;213(2):97–108. doi: 10.1026/0044-3409.213.2.97

[26] Schiefele U , Streblow L , Ermgassen U , et al. Lernmotivation und Lernstrategien als Bedingungen der Studienleistung. Ergebnisse einer Längsschnittstudie. Zeitschrift für pädagogische Psychologie. 2003;17(3/4):185–98. doi: 10.1024//1010-0652.17.34.185

[27] Pires E , Daniel-Filho DA , de Nooijer J , et al. Collaborative learning: elements encouraging and hindering deep approach to learning and use of elaboration strategies. Med Teach. 2020;42(11):1261–9. doi: 10.1080/0142159X.2020.1801996 32780607

[28] Wild K-P . Lernstrategien im Studium. Münster: Waxmann; 2000.

[29] Wild K-P . Individuelle Lernstrategien von Studierenden. Konsequenzen für die Hochschuldidaktik und die Hochschullehre. BzL-Beiträge zur Lehrerinnen-und Lehrerbildung. 2005;23(2):191–206. doi: 10.36950/bzl.23.2.2005.10089

[30] Wild K-P , Schiefele U . Lernstrategien im Studium: Ergebnisse zur Faktorenstruktur und Reliabilität eines neuen Fragebogens. [Learning strategies of university students: Factor structure and reliability of a new questionnaire] Zeitschrift für Differentielle und Diagnostische Psychologie. 1994;15:185–200.

[31] Boerner S , Seeber G , Keller H , et al. Lernstrategien und Lernerfolg im studium. Zeitschrift für Entwicklungspsychologie und pädagogische Psychologie. 2005;37(1):17–26. doi: 10.1026/0049-8637.37.1.17

[32] Boekaerts M . Self-regulated learning: a new concept embraced by researchers, policy makers, educators, teachers, and students. Learning and Instruction. 1997;7(2):161–86. doi: 10.1016/S0959-4752(96)00015-1

[33] Dignath C , Veenman MVJ . The role of direct strategy instruction and indirect activation of self-regulated Learning—Evidence from classroom observation studies. Educational Psychology Review. 2021;33(2):489–533. doi: 10.1007/s10648-020-09534-0

[34] Anthonysamy L , Koo A-C , Hew S-H . Self-regulated learning strategies and non-academic outcomes in higher education blended learning environments: a one decade review. Education and Information Technologies. 2020;25(5):3677–704. doi: 10.1007/s10639-020-10134-2

[35] Wang C-H , Shannon DM , Ross ME . Students’ characteristics, self-regulated learning, technology self-efficacy, and course outcomes in online learning. Distance education. 2013;34(3):302–23. doi: 10.1080/01587919.2013.835779

[36] Li K . MOOC learners’ demographics, self-regulated learning strategy, perceived learning and satisfaction: a structural equation modeling approach. Computers & Education. 2019;132:16–30. doi: 10.1016/j.compedu.2019.01.003

[37] Wissing F . Nationaler kompetenzbasierter Lernzielkatalog Medizin und Zahnmedizin (NKLM/NKLZ). National Competency-Based Learning Objective Catalogue for Dental and Human Medicine]. Bundesgesundheitsblatt Gesundheitsforschung Gesundheitsschutz. 2018;61(2):170. doi: 10.1007/s00103-018-2688-0 29335744

[38] Erschens R , Schröpel C , Herrmann-Werner A , et al. The mediating role of self-efficacy in the relationship between past professional training and burnout resilience in medical education: a multicentre cross-sectional study. BMC Med Educ. 2024;24(1):875. doi: 10.1186/s12909-024-05854-9 39143612 PMC11323524

[39] Schröpel C , Festl-Wietek T , Herrmann-Werner A , et al. Professional and academic pre-qualifications, career preferences and aspirations in working as a rural doctor. Front Med (Lausanne). 2025;12:1566303. doi: 10.3389/fmed.2025.1566303 40703274 PMC12284000

[40] Quotenmodell des Zulassungsverfahrens. Stiftung für Hochschulzulassung; 2026. Available from: https://hochschulstart.de/informieren-planen/verfahrensdetails/quotenmodell-des-zv

[41] Westermann R , Heise E , Spies K . FB-SZ-K-Kurzfragebogen zur Erfassung der Studienzufriedenheit [Verfahrensdokumentation, Fragebogen und Erläuterungen zum Fragebogen] In: Leibniz-Institut für PsychologieHrsg. (ZPID) (Hrsg); 2018.

[42] Klingsieck KB . Kurz und knapp–die Kurzskala des Fragebogens „Lernstrategien im studium (LIST). Zeitschrift für pädagogische Psychologie. 2019. doi: 10.1024/1010-0652/a000230

[43] Rosseel Y . Lavaan: an R package for structural equation modeling. J Stat Softw. 2012;48(1):1–36. doi: 10.18637/jss.v048.i02

[44] Hu Lt , Bentler PM . Cutoff criteria for fit indexes in covariance structure analysis: conventional criteria versus new alternatives. Structural equation modeling: a multidisciplinary journal. 1999;6(1):1–55.

[45] Spörer N , Brunstein JC . Strategien der Tiefenverarbeitung und Selbstregulation als Prädiktoren von Studienzufriedenheit und Klausurleistung. Psychologie in Erziehung und Unterricht. 2005;52(2):127–37.

[46] Demirören M , Turan S , Öztuna D . Medical students’ self-efficacy in problem-based learning and its relationship with self-regulated learning. Med Educ Online. 2016;21(1):30049. doi: 10.3402/meo.v21.30049 26987386 PMC4796725

[47] Theobald M . Self-regulated learning training programs enhance university students’ academic performance, self-regulated learning strategies, and motivation: a meta-analysis. Contemp Educ Psychol. 2021;66:101976. doi: 10.1016/j.cedpsych.2021.101976

[48] Dresel M , Schmitz B , Schober B , et al. Competencies for successful self-regulated learning in higher education: structural model and indications drawn from expert interviews. Studies in Higher Education. 2015;40(3):454–70. doi: 10.1080/03075079.2015.1004236

[49] Cohen MT . The importance of self-regulation for college student learning. Coll Stud J. 2012;46(4):892–902.

[50] van Houten‐Schat MA , Berkhout JJ , Van Dijk N , et al. Self‐regulated learning in the clinical context: a systematic review. Med Educ. 2018;52(10):1008–15. doi: 10.1111/medu.13615 29943415 PMC6175376

[51] Feraco T , Casali N , Ganzit E , et al. Adaptability and emotional, behavioural and cognitive aspects of self‐regulated learning: direct and indirect relations with academic achievement and life satisfaction. Br J Educ Psychol. 2023;93(1):353–67. doi: 10.1111/bjep.12560 36325619

[52] Nabizadeh S , Hajian S , Sheikhan Z , et al. Prediction of academic achievement based on learning strategies and outcome expectations among medical students. BMC Med Educ. 2019;19:1–11. doi: 10.1186/s12909-019-1527-9 30953500 PMC6451267

[53] Zimmerman BJ . Self-regulated learning and academic achievement: an overview. Educational Psychologist. 1990;25(1):3–17. doi: 10.1207/s15326985ep2501_2

[54] Zavlin D , Jubbal KT , Noé JG , et al. A comparison of medical education in Germany and the United States: from applying to medical school to the beginnings of residency. Ger Med Sci. 2017;15. doi: 10.3205/000256 PMC5617919 29051721

[55] Kroher M , Beuße M , Isleib S , et al. Die Studierendenbefragung in Deutschland: 22. Sozialerhebung: die wirtschaftliche und soziale Lage der Studierenden in Deutschland 2021. 2023.

[56] Haider SI , Ahmed F , Pasha H , et al. Life satisfaction, resilience and coping mechanisms among medical students during COVID-19. PLoS One. 2022;17(10):e0275319. doi: 10.1371/journal.pone.0275319 36197934 PMC9534406

[57] Gurpinar E , Alimoglu MK , Mamakli S , et al. Can learning style predict student satisfaction with different instruction methods and academic achievement in medical education? Adv Physiol Ed. 2010;34(4):192–6. doi: 10.1152/advan.00075.2010 21098386

[58] Tessema MT , Ready KJ , Astani M . Does part-time job affect college students’ satisfaction and academic performance (GPA)? the case of a mid-sized public university. International Journal of Business Administration. 2014;5(2):50–9. doi: 10.5430/ijba.v5n2p50

[59] Lauterjung M-L , Ehlers C , Guntinas-Lichius O . PJplus - a project improving practical training during the final year of medical education. Z Evid Fortbild Qual Gesundhwes. 2021;164:70–8. doi: 10.1016/j.zefq.2021.05.009 34253478

[60] Erschens R , Herrmann–Werner A , Keifenheim KE , et al. Differential determination of perceived stress in medical students and high-school graduates due to private and training-related stressors. PLoS One. 2018;13(1):e0191831. doi: 10.1371/journal.pone.0191831 29385180 PMC5792003

[61] Cant R , Gazula S , Ryan C . Predictors of nursing student satisfaction as a key quality indicator of tertiary students' education experience: an integrative review. Nurse Educ Today. 2023;126:105806. doi: 10.1016/j.nedt.2023.105806 37060777

[62] Green HJ , Hood M , Neumann DL . Predictors of student satisfaction with university psychology courses: a review. Psychology Learning & Teaching. 2015;14(2):131–46. doi: 10.1177/1475725715590959

[63] Huang P-H , Velan G , Smith G , et al. What impacts students’ satisfaction the most from Medicine student experience questionnaire in Australia: a validity study. J Educ Eval Health Prof. 2023;20:2. doi: 10.3352/jeehp.2023.20.2 36872423 PMC9986309

